# Supplementary material for: Proteogenomic discovery of neoantigens facilitates personalized multi-antigen targeted T cell immunotherapy for brain tumors
Source: Nat Commun. 2021 Nov 18;12:6689. doi: 10.1038/s41467-021-26936-y (PMC8602676; doi:10.1038/s41467-021-26936-y)
Supplement: Supplementary file 2 — Description of Additional Supplementary Files [file 41467_2021_26936_MOESM2_ESM.docx]

**Title:** Supplementary Data 1.

**Description:** Clinical and tumor sample information.

**Title:** Supplementary Data 2.

**Description:** Summary of medulloblastoma tumor proteomic data.

**Title:** Supplementary Data 3.

**Description:** List of novel peptides detected in medulloblastoma tumors.

**Title:** Supplementary Data 4.

**Description:** Patient 7316-3778 peptides used for T cell stimulation

**Title:** Supplementary Data 5.

**Description:** Top 10 clonotypes within the 7316-3778 TSAT population

**Title:** Supplementary Data 6.

**Description:** Summary of medulloblastoma cell line proteomic data.

**Title:** Supplementary Data 7.

**Description:** List of novel peptides detected in medulloblastoma cell lines.

**Title:** Supplementary Data 8.

**Description:** HLA typing of MB002 and D556 cell lines and healthy donors used in this study.

**Title:** Supplementary Data 9.

**Description:** MB002 and D556 cell line peptides used for T cell stimulation.

**Title:** Supplementary Data 10.

**Description:** Antibodies used in this study.
