# Supplementary material for: Proteogenomic discovery of neoantigens facilitates personalized multi-antigen targeted T cell immunotherapy for brain tumors
Source: Nat Commun. 2021 Nov 18;12:6689. doi: 10.1038/s41467-021-26936-y (PMC8602676; doi:10.1038/s41467-021-26936-y)
Supplement: Supplementary file 14 — Reporting Summary [file 41467_2021_26936_MOESM14_ESM.pdf]

nature research

Corresponding author(s): Brian R. Rood
Last updated by author(s): Oct 21, 2021

Reporting Summary

Nature Research wishes to improve the reproducibility of the work that we publish. This form provides structure for consistency and transparency in reporting. For further information on Nature Research policies, see our Editorial Policies and the Editorial Policy Checklist.

Statistics

For all statistical analyses, confirm that the following items are present in the figure legend, table legend, main text, or Methods section.

n/a	Confirmed	
☐	☒	The exact sample size (*n*) for each experimental group/condition, given as a discrete number and unit of measurement
☐	☒	A statement on whether measurements were taken from distinct samples or whether the same sample was measured repeatedly
☐	☒	The statistical test(s) used AND whether they are one- or two-sided<br>*Only common tests should be described solely by name; describe more complex techniques in the Methods section.*
☒	☐	A description of all covariates tested
☒	☐	A description of any assumptions or corrections, such as tests of normality and adjustment for multiple comparisons
☐	☒	A full description of the statistical parameters including central tendency (e.g. means) or other basic estimates (e.g. regression coefficient) AND variation (e.g. standard deviation) or associated estimates of uncertainty (e.g. confidence intervals)
☐	☒	For null hypothesis testing, the test statistic (e.g. *F*, *t*, *r*) with confidence intervals, effect sizes, degrees of freedom and *P* value noted<br>*Give P values as exact values whenever suitable.*
☒	☐	For Bayesian analysis, information on the choice of priors and Markov chain Monte Carlo settings
☒	☐	For hierarchical and complex designs, identification of the appropriate level for tests and full reporting of outcomes
☒	☐	Estimates of effect sizes (e.g. Cohen's *d*, Pearson's *r*), indicating how they were calculated

Our web collection on statistics for biologists contains articles on many of the points above.

Software and code

Policy information about availability of computer code

Data collection

- RNA-seq and WGS: Cells line RNA-seq data were generated on Illumina HiSeq platform with 2x150 bp read length to an average of 200M reads per sample by GENEWIZ. Data acquisition software: HCS 2.2.58.
- Proteomics: The peptide mixtures from each fraction were sequentially analyzed by LC-MS/MS using the nano-LC system (Easy nLC1000) connected to a Q Exactive HF mass spectrometer (Thermo Scientific). Data acquisition software: Xcalibur 4.3
- Flow Cytometry: acquisition was performed on a CytoFlex S (Beckman Coulter) . Data acquisition software: CytExpert version 2.2.0.97.

Data analysis

The following softwares have been used in this manuscript:
Flow cytometry:
FlowJo version 10.7.2 analysis software (BD Biosciences, Ashland, OR)
WGS and RNA-seq:
bwa (v.0.7.17) , PicardTools (v2.18.1), GATK(v3.8), GATK-Mutect2 (v.4.0.2.1) STAR (v.2.5.1), Defuse (v.0.6.1) ericscript(v.1) and STAR-Fusion (v.0.8.0)
TCR-sequencing:
TCR-seq was analyzed using the immunoSEQ ANALYZER v3.0 from Adaptive Technologies
Proteomics:
Thermo Proteome Discoverer Software (version 2.3), R(v3.6), the R package CustomProDB (v1.32), SSRCalc tool (versionQ, http://hs2.proteome.ca/SSRCalc/SSRCalcQ.html). AutoRT (https://github.com/bzhanglab/AutoRT), blastp (ncbi-blast-2.10.0)

For manuscripts utilizing custom algorithms or software that are central to the research but not yet described in published literature, software must be made available to editors and reviewers. We strongly encourage code deposition in a community repository (e.g. GitHub). See the Nature Research guidelines for submitting code & software for further information.

Data

Policy information about availability of data

All manuscripts must include a data availability statement. This statement should provide the following information, where applicable:

- Accession codes, unique identifiers, or web links for publicly available datasets
- A list of figures that have associated raw data
- A description of any restrictions on data availability

The genomic CBTN data (RNA-seq and WGS) is this study are publicly available. The genomic CBTN data used in this study are available through the public project "Pediatric Brain Tumor Atlas: CBTTC" on the Kids First Data Resource Portal [https://kidsfirstdrc.org/] and Cavatica [https://cbtn.org/]. The data is available under restricted access. Access can be obtained by submitting the CBTN Data Access form found in the Kids First Data Resource Portal [https://kidsfirstdrc.org/] or CBTN web [https://cbtn.org/]. Additionally, cell line RNA-seq reads are available in the Sequence Read Archive (SRA) under the accession number SRP276163 [https://trace.ncbi.nlm.nih.gov/Traces/sra/?study=SRP276163]. The newly generated mass spectrometry proteomics data have been deposited to the ProteomeXchange Consortium via the PRIDE65 partner repository with the dataset identifier PXD029082 [https://www.ebi.ac.uk/pride/archive/projects/PXD029082]. TCR-seq data are freely accessible through ImmuneAccess [https://clients.adaptivebiotech.com/pub/rivero-hinojosa-2021-nc]. This work used previously generated genomic data from GTEX project obtained from dbGaP under accession number phs000424.v2.p1 [https://www.ncbi.nlm.nih.gov/projects/gap/cgi-bin/study.cgi?study_id=phs000424.v2.p1]. This work used the UniProt human proteome database UP000005640 [https://www.uniprot.org/proteomes/UP000005640]. This work used the previously generated proteomic dataset deposited on PRIDE under the accession number PXD006109 [https://www.ebi.ac.uk/pride/archive/projects/PXD006109]. The remaining data are available within the Article, Supplementary Information or Source Data file.

Field-specific reporting

Please select the one below that is the best fit for your research. If you are not sure, read the appropriate sections before making your selection.

☒ Life sciences ☐ Behavioural & social sciences ☐ Ecological, evolutionary & environmental sciences

For a reference copy of the document with all sections, see nature.com/documents/nr-reporting-summary-flat.pdf

Life sciences study design

All studies must disclose on these points even when the disclosure is negative.

Sample size	No statistical methods were used to predetermine the experimental sample size. The sample size was determined based on patient sample availability. The analyses performed were descriptive rather than comparative in nature.
Data exclusions	No data were excluded.
Replication	For all applicable figures, two or three independent experiments were performed and all attempts at replicating observation were successful. The replication number is indicated in the legend of corresponding figures and the methods.
Randomization	Randomization is not applicable to this study as there is no comparative analysis.
Blinding	No blinding was applied experiments during data collection as all data are quantitative and not subjected to investigator bias. In addition, there were no comparative analyses performed.

Reporting for specific materials, systems and methods

We require information from authors about some types of materials, experimental systems and methods used in many studies. Here, indicate whether each material, system or method listed is relevant to your study. If you are not sure if a list item applies to your research, read the appropriate section before selecting a response.

Materials & experimental systems

n/a	Involved in the study	
☐	☒	Antibodies
☐	☒	Eukaryotic cell lines
☒	☐	Palaeontology and archaeology
☒	☐	Animals and other organisms
☐	☒	Human research participants
☒	☐	Clinical data
☒	☐	Dual use research of concern

Methods

n/a	Involved in the study	
☒	☐	ChIP-seq
☐	☒	Flow cytometry
☒	☐	MRI-based neuroimaging

Antibodies

Antibodies used	Antibodies used are listed in Supplementary Table S10 with extensive details.

FLOW CITOMETRY PANELS
PANEL:Activation & Function
- FITC CD3, Clone:UCHT1. Biolegend, San Diego, CA, USA
- PE TNF-a (IC) , Clone:REA656. Miltenyi Biotech, Gladbach, Germany
- PE-Cy7 CD223 (LAG3), Clone:11C3C65. BioLegend, San Diego, CA, USA
- APC CTLA4 (IC), Clone:REA1003. Miltenyi Biotech, Gladbach, Germany
- Ax700 IFN-γ (IC), Clone:4S.B3. eBioScience, San Diego, CA, USA
- APC-Fire750 PD-1, Clone: 29F.1A12. BioLegend, San Diego, CA, USA
- BV421 CD8, Clone:RPA-T8. BioLegend, San Diego, CA, USA
- Live/Dead Aqua. ThermoFisher Scientific, MA, USA
- BV605 CD4, Clone: OKT4. BioLegend, San Diego, CA, USA
- BV650 CD366 (TIM3), Clone:F38-2E2. BioLegend, San Diego, CA, USA

PANEL: Memory & Differentiation
- FITC CD3, Clone:UCHT1. Biolegend, San Diego, CA, USA
- PE-Vio770 CD45RO, Clone: REA611. Miltenyi Biotech, Gladbach, Germany
- APC CCR7, Clone:REA108. Miltenyi Biotech, Gladbach, Germany
- APC-Fire750 CD27, Clone: O323. BioLegend, San Diego, CA, USA
- BV421 CD8, Clone:RPA-T8. BioLegend, San Diego, CA, USA
- Live/Dead Aqua. ThermoFisher Scientific, MA, USA
- BV605 CD4, Clone:OKT4. BioLegend, San Diego, CA, USA
- BV650 CD62L, Clone: DREG-56, BioLegend, San Diego, CA, USA

PANEL: Populations
- FITC CD3, Clone: UCHT1. Biolegend, San Diego, CA, USA
- PE CD127 (IL-7Rα), Clone:A019D5. Biolegend, San Diego, CA, USA
- APC TCRγδ, Clone:REA591. Miltenyi Biotech, Gladbach, Germany
- AlexaFluor700 CD25, Clone: BC96. BioLegend, San Diego, CA, USA
- APC-Fire750 CD16, Clone: 3G8. Biolegend, San Diego, CA, USA
- BV421 CD8 Clone: RPA-T8. BioLegend, San Diego, CA, USA
- Live/Dead Aqua. ThermoFisher Scientific, MA, USA
- BV605 CD4, Clone: OKT4. BioLegend, San Diego, CA, USA
- BV650 CD56, Clone: HCD56. BioLegend, San Diego, CA, USA

PANEL: Cytotoxicity
- Cell Trace Violet. ThermoFisher, USA
- Live/Dead Green, Clone: A019D5. ThermoFisher, USA
- BV785 CD3, Clone: OKT4. Biolegend, San Diego, CA, USA

ADITIONAL ANTIBODIES
Anti-IFN-γ Enzyme-Linked Immuno-spot (ELISpot) Assay
- Capture: anti-human IFN-γ mAb. Clone: 1-D1K, purified. Mabtech,Cincinnati, OH, USA
- Detection: Biotinylated-anti-human IFN-γ mAb. Clone: 7-B6-1. Mabtech,Cincinnati, OH, USA
HLA Blocking antibodies
-Monoclonal Mouse Anti-Human HLA-ABC Antigen, Clone W6/32. Agilent Dako,Santa Clara, CA, US
-Monoclonal Mouse Anti-Human HLA-DP, DQ, DR Antigen, Clone CR3/43. Agilent Dako,Santa Clara, CA, US

Validation

The antibodies used have been extensively used in the field and validated by the manufacturing companies
-FITC CD3, Clone:UCHT1. Biolegend, San Diego, CA, USA
https://www.biolegend.com/en-us/products/fitc-anti-human-cd3-antibody-863?GroupID=BLG5900
-PE TNF-a (IC) , Clone:REA656. Miltenyi Biotech, Gladbach, Germany
https://www.miltenyibiotec.com/US-en/products/tnf-a-antibody-anti-human-reafinity-rea656.html#pe:30-tests-in-60-ul
-PE-Cy7 CD223 (LAG3), Clone:11C3C65. BioLegend, San Diego, CA, USA
https://www.biolegend.com/en-us/search-results/pe-cyanine7-anti-human-cd223-lag-3-antibody-13551
-APC CTLA4 (IC), Clone:REA1003. Miltenyi Biotech, Gladbach, Germany
https://www.miltenyibiotec.com/US-en/products/cd152-antibody-anti-human-reafinity-rea1003.html#apc:100-tests-in-200-ul
-Ax700 IFN-γ (IC), Clone:4S.B3. eBioScience, San Diego, CA, USA
https://www.thermofisher.com/antibody/product/IFN-gamma-Antibody-clone-4S-B3-Monoclonal/56-7319-42
-APC-Fire750 PD-1, Clone: 29F.1A12. BioLegend, San Diego, CA, USA
https://www.biolegend.com/en-us/products/apc-fire-750-anti-mouse-cd279-pd-1-antibody-13637?GroupID=BLG7930
-BV421 CD8, Clone:RPA-T8. BioLegend, San Diego, CA, USA
https://www.bdbiosciences.com/us/reagents/research/antibodies-buffers/immunology-reagents/anti-human-antibodies/cell-surface-antigens/bv421-mouse-anti-human-cd8-rpa-t8/p/562428
-Live/Dead Aqua. ThermoFisher Scientific, MA, USA
https://www.thermofisher.com/order/catalog/product/L34957#/L34957
-BV605 CD4, Clone: OKT4. BioLegend, San Diego, CA, USA
https://www.biolegend.com/en-us/explore-new-products/brilliant-violet-605-anti-human-cd4-antibody-7820
-BV650 CD366 (TIM3), Clone:F38-2E2. BioLegend, San Diego, CA, USA

https://www.biolegend.com/en-us/search-results/brilliant-violet-650-anti-human-cd366-tim-3-antibody-12008
-PE-Vio770 CD45RO, Clone: REA611. Miltenyi Biotech, Gladbach, Germany
https://www.miltenyibiotec.com/US-en/products/cd45ro-antibody-anti-human-reafinity-rea611.html#gref
-APC CCR7, Clone:REA108. Miltenyi Biotech, Gladbach, Germany
https://www.miltenyibiotec.com/US-en/products/cd197-ccr7-antibody-anti-human-reafinity-rea108.html#apc:30-tests-in-60-ul
-APC-Fire750 CD27, Clone: O323. BioLegend, San Diego, CA, USA
https://www.biolegend.com/fr-ch/products/apc-fire-750-anti-human-cd27-antibody-13716
-BV650 CD62L, Clone: DREG-56, BioLegend, San Diego, CA, USA
https://www.biolegend.com/en-us/search-results/brilliant-violet-650-anti-human-cd62l-antibody-8059
-PE CD127 (IL-7Rα), Clone:A019D5. Biolegend, San Diego, CA, USA
https://www.biolegend.com/en-us/products/pe-anti-human-cd127-il-7ralpha-antibody-7094
-APC TCRγδ, Clone:REA591. Miltenyi Biotech, Gladbach, Germany
-anti-human IFN-γ mAb. Clone: 1-D1K, purified. Mabtech,Cincinnati, OH, USA
https://www.miltenyibiotec.com/US-en/products/tcrg-d-antibody-anti-human-reafinity-rea591.html#gref
-AlexaFluor700 CD25, Clone: BC96. BioLegend, San Diego, CA, USA
https://www.biolegend.com/en-us/products/alexa-fluor-700-anti-human-cd25-antibody-3418?GroupID=BLG7917
-APC-Fire750 CD16, Clone: 3G8. Biolegend, San Diego, CA, USA
https://www.biolegend.com/en-us/search-results/apc-fire-750-anti-human-cd16-antibody-13192
-BV421 CD8 Clone: RPA-T8. BioLegend, San Diego, CA, USA
https://www.biolegend.com/en-us/search-results/brilliant-violet-421-anti-human-cd8a-antibody-7152
-BV650 CD56, Clone: HCD56. BioLegend, San Diego, CA, USA
https://www.biolegend.com/en-us/search-results/brilliant-violet-650-anti-human-cd56-ncam-antibody-8780?GroupID=BLG15664
-Cell Trace Violet. ThermoFisher, USA
https://www.thermofisher.com/order/catalog/product/C34557#/C34557
-Live/Dead Green, Clone: A019D5. ThermoFisher, USA
https://www.thermofisher.com/order/catalog/product/L34970#/L34970
- Capture: anti-human IFN-γ mAb. Clone: 1-D1K, purified. Mabtech,Cincinnati, OH, USA
https://www.mabtech.com/products/anti-human-ifn-gamma-antibody-1-d1k-purified-3420-3
- Detection: Biotinylated-anti-human IFN-γ mAb. Clone: 7-B6-1. Mabtech,Cincinnati, OH, USA
https://www.mabtech.com/products/anti-human-ifn-gamma-antibody-7-b6-1-biotinylated-3420-6
-Monoclonal Mouse Anti-Human HLA-ABC Antigen, Clone W6/32. Agilent Dako,Santa Clara, CA, US
https://www.agilent.com/store/productDetail.jsp?catalogId=M073601-2
-Monoclonal Mouse Anti-Human HLA-DP, DQ, DR Antigen, Clone CR3/43. Agilent Dako,Santa Clara, CA, US
https://www.agilent.com/store/productDetail.jsp?catalogId=M077501-2

Eukaryotic cell lines

Policy information about cell lines

Cell line source(s)	MB002 and MB004 were gifts from Y.J. Cho (Oregon Health and Science University, Portland, OR, United States). D556 cell was a gift from Darell Bigner (Duke, University School of Medicine). D283 was obtained from ATCC (https://www.atcc.org/products/htb-185)
Authentication	Cell lines have been authenticate by STR Profiling at The Genetic Resources Core Facility of Johns Hopkins University, School of Medicine, McKusick- Nathans Department of Genetic Medicine.
Mycoplasma contamination	The cell lines used were confirmed negative for mycoplasma contamination
Commonly misidentified lines (See ICLAC register)	None of the cell lines used in this manuscript are listed in the ICLAC Database of Cross-contaminated or Misidentified Cell Lines

Human research participants

Policy information about studies involving human research participants

Population characteristics	Tumor samples were obtained from either the CNH or CBTN tissue repositories and were de-identified by those entities. The investigators were only privy to the tumor diagnosis and no identifying or demographic details.
Recruitment	Subjects themselves were not recruited. Rather tissue samples were requested from the Children's National Medical Center and Children's Brain Tumor Network tissue repositories on the basis of diagnosis and availability. No other parameters were placed upon sample acquisition.
Ethics oversight	The subjects (or their parents) whose tumor material was deposited in the tissue banks were consented by the Children's National Medical Center Institutional Review Board and Children's Brain Tumor Network approved protocol.

Note that full information on the approval of the study protocol must also be provided in the manuscript.

Flow Cytometry

Plots

Confirm that:

- [x] The axis labels state the marker and fluorochrome used (e.g. CD4-FITC).
- [x] The axis scales are clearly visible. Include numbers along axes only for bottom left plot of group (a 'group' is an analysis of identical markers).
- [x] All plots are contour plots with outliers or pseudocolor plots.
- [x] A numerical value for number of cells or percentage (with statistics) is provided.

Methodology

Sample preparation	Phenotyping: Tumor antigen-experienced T cells expanded by 3 stimulations with dendritic cells w ere resuspended at 10e6/mL & 100 uL plated in 96 well U-bottom plates (1e6/well) for surface staining. For intracellular cytokine detection cells were stimulated with peptides for 2 hours and Brefeldin A added for a further 4 hours prior to surface & intracellular staining. Cytotoxicity: tumor cells were first plated in 96 well U-bottom plates (1e4/well) followed by expanded T cells (1e5/well). Co-cultures were incubated for up to 96 hours, with 1 plate removed at each time point for surface staining. Cells were labeled with FcR block & viability dye (1 wash) then surface antibodies (15 mins, room temp, dark). Following 1 wash cells were fixed then permeabilized for intracellular AB staining (30 mins, 4C, dark). Acquisition occurred within 24hours
Instrument	Beckman Coulter CytoFlex S
Software	FlowJo Version 10.7.2
Cell population abundance	N/A No sorting was conducted in these experiments
Gating strategy	Phenotype: boundaries of positivity determined by unstained, single stains & FMOs during panel development<br>Panel 1<br>Flow stability & exclusion of sub-cellular events: Time vs FSC-H (eliminate<br>Cells: FSC-A v SSC-A<br>Pulse Geometry (Single cells): FSC-H v FSC-A<br>Viability (Live/Dead Aqua) vs CD3-FITC<br>Live CD3+ -> CD4+ v CD8+<br>CD4+ -> CD25 v CD127 (putative Treg)<br>CD3+ -> CD16 v CD56 (NKT)<br>CD3+CD4-CD8- -> TCRyd v CD56 (TCRyd)<br>CD3- -> CD16 v CD56 (NK)<br><br>Panel 2<br>Flow stability & exclusion of sub-cellular events: Time vs FSC-H<br>Cells: FSC-A v SSC-A<br>Pulse Geometry (Single cells): FSC-H v FSC-A<br>Viability (Live/Dead Aqua) vs CD3-FITC<br>Live CD3+ -> CD4+ v CD8+<br>CD4+/CD8+ -> CD45RO+/-<br>CD45RO+ -> CD62L vs CCR7 (Tcm, Tem)<br>CD45RO- -> CD62L vs CCR7 (Tn, Teff) -> CD95 v CD127 (Tscm)<br><br>Panel 3<br>Flow stability & exclusion of sub-cellular events: Time vs FSC-H<br>Cells: FSC-A v SSC-A<br>Pulse Geometry (Single cells): FSC-H v FSC-A<br>Viability (Live/Dead Aqua) vs CD3-FITC<br>Live CD3+ -> CD4+ v CD8+ -> TNF-a v IFN-y<br><br>Cytotoxicity<br>Flow stability & exclusion of sub-cellular events: Time vs FSC-H<br>Viability (Live/Dead Green) vs FSC-H<br>No pulse geometry applied due to co-culture of 2 cell types of widely varying sizes & synapses of T & tumor cells. Singles gate not appropriate in this scenario.<br>CD3 BV785 (T cells) v Cell Trace Violet (tumor targets)

- [x] Tick this box to confirm that a figure exemplifying the gating strategy is provided in the Supplementary Information.
